# Reactive oxygen species mediate bioeffects of static magnetic field via impairment of long-chain fatty acid degradation in *Escherichia coli*

**DOI:** 10.3389/fmicb.2025.1586233

**Published:** 2025-06-25

**Authors:** Haodong Li, Yanwen Fang, Jirong Huang

**Affiliations:** ^1^Shanghai Key Laboratory of Plant Molecular Sciences, College of Life Sciences, Shanghai Normal University, Shanghai, China; ^2^Heye Health Industrial Research Institute of Zhejiang Heye Health Technology, Anji, Zhejiang, China

**Keywords:** static magnetic field (SMF), reactive oxygen species, long-chain fatty acid, oxidative stress, *Escherichia coli*

## Abstract

Static magnetic fields (SMF) have been shown to influence bacterial growth via reactive oxygen species (ROS). However, the underlying mechanisms remain poorly understood. This study investigated the role of ROS in mediating the growth inhibitory effect of SMF on *Escherichia coli*. We demonstrated that exposure of bacteria to a 250 mT SMF significantly elevates ROS level, as confirmed by a chemical fluorescent probe, electron paramagnetic resonance (EPR) spectroscopy, and a genetically engineered redox biosensor. Transcriptomic analysis revealed that SMF- and hydrogen peroxide (H_2_O_2_) treatments share a set of common differentially expressed genes (DEGs), particularly those involved in long chain fatty acid metabolism, the tricarboxylic acid (TCA) cycle, and defense mechanisms against ROS stress. Specifically, SMF downregulates the expression of the *fadD* gene, impairing long-chain fatty acid (LCFA) degradation, which is critical for bacterial growth. Interestingly, overexpression of the superoxide dismutase gene *SodB* alleviated SMF-induced growth inhibition, highlighting the pivotal role of ROS in this process. Taken together, our findings provide novel insights into the molecular mechanism by which oxygen serves as a magnetic target, triggering ROS signaling, and enabling bacteria to adapt to SMF exposure.

## Introduction

Reactive oxygen species (ROS) are a group of chemically reactive molecules derived from oxygen (O_2_), including superoxide anions (${\text{O}}_{2}^{•-}$), hydrogen peroxide (H_2_O_2_), and hydroxyl radicals (•OH). These species are naturally generated during cellular metabolism and play important roles in maintaining cellular homeostasis via catalyzing biochemical reactions and initiating signaling pathways. However, excessive ROS accumulation can lead to oxidative stress, causing irreversible damage to cellular macromolecules such as DNA, proteins, and lipids. For instance, long-chain fatty acid (LCFA), which are essential for bacterial cell membrane synthesis, lipid storage, and signaling, are susceptible to ROS-mediated alterations. ROS can modulate LCFA biosynthesis by oxidizing thiol groups in key enzymes like acetyl-CoA carboxylase and fatty acid synthase, thereby influencing their activity (Forrester et al., 2018). Thus, ROS exhibit a dual role in cellular physiology, acting as both signaling molecules and damaging agents, depending on the delicate balance between their production and scavenging within specific temporal and spatial contexts.

In bacteria systems, ROS are primarily generated from electron transport chain (ETC) located in the plasma membrane during oxidative phosphorylation, which is performed by a series of protein complexes including NADH dehydrogenase (complex I), succinate dehydrogenase (complex II), cytochrome *bc*1 complex (complex III), and cytochrome *c* oxidase (complex IV). Electron leakage, particularly from complex I and complex III, results in the partial reduction of molecular oxygen to form ${\text{O}}_{2}^{•-}$. These ${\text{O}}_{2}^{•-}$ are subsequently converted into H_2_O_2_, a less oxidative species by superoxide dismutase (SOD). In the presence of iron (Fe^2+^), H_2_O_2_ can be further turned into the highly reactive species •OH via the Fenton reaction. In addition, ROS can be generated by various flavoproteins, such as NADPH oxidases and dehydrogenases, as well as by external stressors like ultraviolet radiation and antibiotics (Imlay, 2003). To counteract ROS-induced damage, bacteria have evolved a sophisticated antioxidant defense system, comprising enzymatic antioxidants such as SOD, which dismutates ${\text{O}}_{2}^{•-}$ into O_2_ and H_2_O_2_; catalase (CAT) and glutathione peroxidase (GPX), which covert H_2_O_2_ into O_2_ and H_2_O; and non-enzymatic antioxidants like glutathione (GSH) and vitamins C and E. Furthermore, ROS function as signaling molecules in bacteria, sensed by transcription regulators such as OxyR and SoxRS, which activate the expression of diverse antioxidant genes in response to H_2_O_2_ and ${\text{O}}_{2}^{•-}$, respectively (Imlay, 2013).

The production of ROS can also be influenced by external factors, including static magnetic fields (SMF), through the radical pair mechanism (RPM; Scaiano et al., 1994; Hore and Mouritsen, 2016; Zadeh-Haghighi and Simon, 2022). According to the RPM, radical pairs can exist in singlet or triplet states, depending on the spin configuration of their unpaired electrons. Singlet states, characterized by antiparallel spins, are more reactive and prone to recombination, whereas triplet states, with parallel spins, are relatively stable. Magnetic fields can affect the energy levels of the triplet state, potentially facilitating transitions to the singlet state. Specifically, SMF may stabilize or destabilize triplet states, thereby influencing the equilibrium between singlet and triplet radical pairs. When the moderate-intensity magnetic field promotes the conversion of the triplet state to the singlet state, it enhances the likelihood of radical recombination, leading to the formation of more reactive species such as hydrogen peroxide and hydroxyl radicals. Consequently, the RPM model predicts that exposure of SMF will increase the average radical concentration, prolong their lifetimes, and enhance the probability of radical reactions with cellular components.

Despite these theoretical predictions, the effects of SMF on ROS accumulation in bacteria remain inconsistent across studies. Some reports indicate that SMF elevates ROS levels, while others suggest a reduction, with outcomes contingent on factors such as magnetic field intensity, exposure duration, microbial species, and experimental conditions (Okano, 2008; Barnes and Greenebaum, 2015). In some cases, the effects of SMF on bacterial growth are restricted within a certain time window, known as the biological window effect (Łebkowska et al., 2018). Given the multifaceted roles of ROS in bacterial growth, stress responses, and pathogenicity, cellular ROS levels are tightly regulated by a complex interplay of redox enzyme activities, membrane integrity, and intracellular signaling pathways. This regulation ensures a precise balance between ROS production and scavenging, underscoring the need for further investigation into the molecular mechanisms underlying SMF-induced ROS modulation.

We previously reported that SMF-inhibited growth of *Escherichia coli* colonies is closely linked to the accumulation of LCFA, and proposed that free radicals generated from LCFA degradation serve as primary target of SMF action, triggering oxidative stress response in bacteria (Li et al., 2022). In this study, we provide further evidence that SMF exposure elevates ROS levels, notably hydrogen peroxide and hydroxyl radicals, likely through RPM. These findings reinforce the critical role of ROS in mediating the biological effects of SMF in bacterial systems, offering new insights into the interplay between magnetic fields, oxidative stress, and cellular metabolism.

## Materials and methods

### Treatment conditions of SMF

Static magnetic field was mainly provided by permanent sintered magnets made from NdFeB containing PrNd (31 wt.%), B (1 wt.%), Fe (67.5 wt.%) and other (0.5 wt.%; Hangzhou Permanent Magnet Group Co., Ltd., Xiaoshan, Hangzhou, China, https://www.china-hpmg.com) in this study. The magnet was a cube with each side having a length of 10 cm. The average magnetic field intensity at the surface of the north and south poles is 300 mT, while the intensity at the bacteria growth plane is 250 mT. For electron paramagnetic resonance (EPR) spectroscopy, a gradient magnetic field with a maximum intensity of 300 mT was applied using a magnetic stand.

### Bacterial stains, plasmids, and culture conditions

The bacterial strains and plasmids used in this study are shown in Supplementary Table S7. The *Escherichia coli* (*E. coli*) TOP10 strain was used as the wild-type strain in the study. Knockout mutants in the TOP10 background were constructed using the CRISPR-Cas9-based genome editing tool pEcCas/pEcgRNA system (Li et al., 2021). *E. coli* strains were routinely cultured aerobically in Luria-Broth (LB) media at 37°C or maintained on LB agar plates, unless otherwise specified. Fatty acid (10 μM) was added to the solid media when they were needed. Strains with empty vector or complement plasmids were inoculated on solid LB media with ampicillin (Amp, 100 μg/mL), and IPTG (50 μM). For colony formation assays, *E. coli* strains were initially cultured in 5 mL liquid LB media at 37°C and 200 rpm overnight and then serially diluted with LB to obtain a series of gradient dilutions, and 10 μL of bacteria solutions was inoculated on solid LB media containing 1.5% bacto-agar. Plates were put on the magnet pole as an SMF treatment or on an iron cube made of the same materials as that with the SMF but without a magnetic field as a control. Bacterial growth was visualized using a stereoscope (SDFPLAPO1xPF, Olympus, Japan) after 10 h of incubation. Colony-forming units (CFUs) were quantified, and colony diameters were measured using ImageJ software. For transcriptome sequencing experiment, bacteria cultures were grown in 5 mL liquid LB media until the optical density (OD) reached 1.0. The cultures were then aliquoted into 24-well plates. For hydrogen peroxide treatment, H_2_O_2_ was added to respective wells and incubated at 37°C for 10 min. For SMF treatment, plates were placed on the magnet cube, and incubated at 37°C for 1 h. Control groups were treated identically but without SMF exposure.

### Measurement of ROS using DCFH-DA

Intracellular ROS levels in *E. coli* were assessed using the oxidant-sensitive fluorescent probe 2′,7′-dichlorodihydrofluorescein diacetate (DCFH-DA). Following 10 h of SMF exposure, bacterial cells were harvested from solid plates, washed with phosphate-buffered saline (PBS), and stained with 1 μg/mL DCFH-DA for 1 h. After two additional washes with PBS, ROS levels were quantified using a CyAn^TM^ ADP flow cytometer (Beckman Coulter, Brea, CA, USA). 8 μM H_2_O_2_ treatment was implemented as the positive control.

### HyPer probe microscopic imaging and analysis

The HyPer redox-sensitive probe was synthesized and cloned into the pEX8 vector. For hydrogen peroxide analysis, after cultured under SMF for 10 h, *E. coli* TOP10 cells transformed with pE-HyPer (WT/pE-HyPer) were collected for fluorescence imaging. Fluorescence imaging was performed using a laser confocal fluorescence microscope (Olympus, Tokyo, FV3000). To ensure reproducibility and accuracy, multiple fields of view were imaged for each sample, and the acquired fluorescence images were analyzed using ImageJ software.

### EPR for measurement of radicals

EPR measurements were performed using a BRUKER EMXPLUS (Bruker, Germany), operating in X-band (9.84 GHz) with the following parameters: sweep width of 100 G, center field of 3505 G, sweep time of 60 s, modulation amplitude of 2 G, modulation frequency of 100 kHz, power attention of 25 dB, and time constant of 5.12 ms.

For bacterial samples, *E. coli* with an OD of 1.0 was inoculated into 1.5 mL centrifuge tubes and then incubated at 37°C for 1 h. Then, 5 μL of 5,5-dimethyl-1-pyrroline N-oxide (DMPO) as capturing agent was mixed with 200 μL of bacterial culture. After ultrasonic treatment for 30 s, 30–40 μL of solution was sampled into the quartz standard sampling tube and the bottom of sampling tube was sealed with wax sealing plate. The quartz standard sampling tube was then placed in EPR specific sample tube for further detection.

Hydroxyl radical was produced using an ultraviolet (UV)/H_2_O_2_ system: 5 μL of DMPO was mixed with 200 μL H_2_O_2_ (10 mM). After vortex mixing and irradiate with ultraviolet lamp for 1 min, 30–40 μL of solution was sampled for further detection. Superoxide was produced using a XO/HX system: XO (xanthine oxidase, 5 mU/mL) was added to 500 mL PBS solution (0.1 M, pH 7.8) containing HX (hypoxanthine, 1 mM), diethylenetriaminepentaacetic acid (DTPA, 1 mM) and DMPO (97 mM). 30–40 μL of solution was sampled for further detection every 2 min.

### Transcriptomic analysis and quantitative PCR

Total RNA was extracted from bacterial samples using the TIANGEN RNA Extraction Kit. RNA quality and quantity were assessed by the NanoPhotometer^®^ spectrophotometer (IMPLEN, Westlake Village, CA, USA) and the RNA Nano 6000 Assay Kit of the Bioanalyzer 2100 system (Agilent Technologies, Santa Clara, CA, USA). RNA-seq libraries were constructed and sequenced according to the methods provided by Novogene Co., Ltd. (Beijing, China). Raw reads in Fastq format were processed to remove those containing the adapter and poly-N and those with low quality, yielding high-quality clean reads for downstream analysis.

The reference genome and gene model annotation files were obtained from the NCBI database (https://www.ncbi.nlm.nih.gov/assembly/, accessed February 2024). Clean reads were aligned to the reference genome using Bowtie2-2.2.3 (Langmead and Salzberg, 2012), and gene expression levels were quantified using HTSeq v0.6.1. Differential expression analysis between SMF-, H_2_O_2_-treated, and control groups (each with four biological replicates) was performed using the DESeq R package (1.18.0), which employs a negative binomial distribution model. The resulting *p* values were adjusted using Benjamini and Hochberg's approach for controlling the false discovery rate. Functional enrichment analysis of differentially expressed genes (DEGs) was conducted using the Kyoto Encyclopedia of Genes and Genomes (KEGG) orthology-based annotation system (KOBAS; Xie et al., 2011).

For validation of RNA-seq results, quantitative real-time PCR (qPCR) was performed. Reverse transcription was carried out using 1 μg of total RNA with the PrimeScript RT reagent Kit With gDNA Eraser (TaKaRa, Dalian, China). qPCR was performed using SYBR Green Master Mix (Yeasen, China) on QuantStudio 3 (Thermo Fisher Scientific, Waltham, MA, USA). The 16S rRNA was used as an internal control, and the primers used are listed in Supplementary Table S6.

### Enzyme assays of SOD-overexpressed strain

The cellular protein content was measured using a bradford reagent, with the content calculated according to the protocol. SOD activity was measured using a Total Superoxide Dismutase Activity Colorimetric Assay Kit, with activity determined from the inhibiting rate of the enzyme to superoxide (${\text{O}}_{2}^{•-}$) produced by xanthine morpholine with xanthine oxidase, and the activity value was calculated using the formula outlined in the SOD assay kit.

## Results

### SMF inhibits bacterial growth by repressing long-chain fatty acid utilization

**Figure 1:**
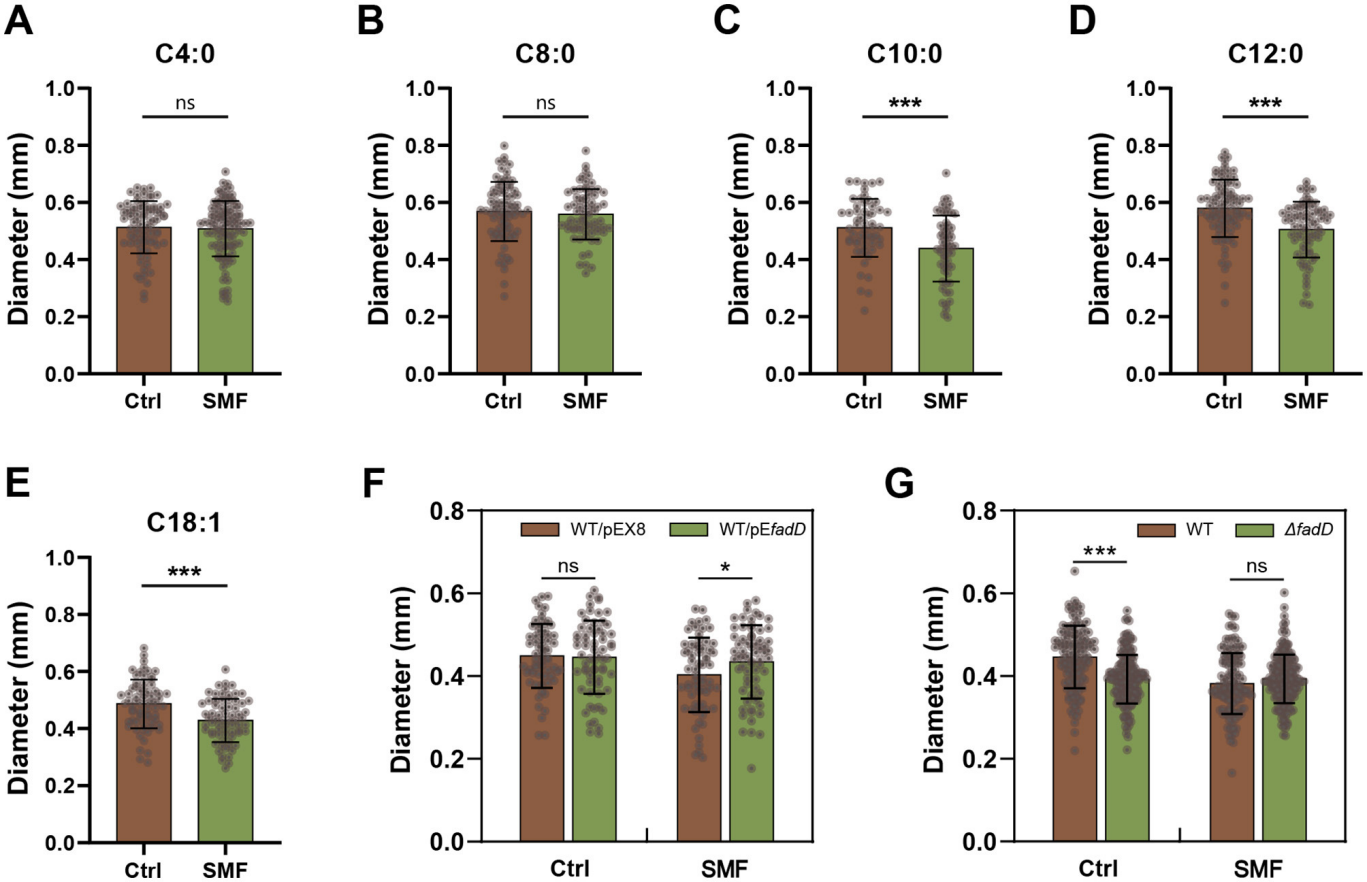
SMF inhibits the growth of *E. coli* colonies through repressing long-chain fatty acid degradation. **(A–E)** Fatty acids with carbon chain shorter than 10 restore the bacterial phenotype in SMF, but fatty acids with carbon chain longer than 10 cannot exert this restorative effect. **(F)** Overexpression of *fadD* restores the SMF-inhibited growth of bacteria. **(G)** Magnetic bioeffect is not observed in the loss-of-function mutant *fadD*. Data are shown as mean ± SD. Statistical analysis was performed by student *t*-test. ^ns^*p* > 0.05; *p < 0.05; ***p < 0.001.

We previously reported that the application of 250 mT SMF significantly inhibits bacterial growth and modifies the carbon source utilization (Li et al., 2022). To identify the specific LCFA responsible for the SMF-induced growth inhibition of *E. coli* colonies, we cultured bacteria on the media supplemented with various fatty acid under 250 mT SMF and geomagnetic field (GMF) conditions. Our results indicated that fatty acid with carbon chains shorter than 10 carbons, but not those with longer chains, effectively restored the bacterial growth under SMF (Figures 1A–E). This observation aligns with the downregulation of the *fadD* gene, which encodes fatty acid-CoA ligase, a key enzyme in the activation of LCFA (Iram and Cronan, 2006; Li et al., 2022; Pavoncello et al., 2022). These findings suggest that SMF inhibits bacterial growth by impairing FadD-mediated LCFA degradation. To further validate this hypothesis, we constructed transgenic *E. coli* strains overexpressing *fadD* (WT/pE*fadD*) and knockout strains Δ*fadD*. Overexpression of *fadD* resulted in a significant increase in colony diameter compared to the control (WT/pEX8) under SMF (Figure 1F). Consistently, deletion of *fadD* led to bacterial growth inhibition even under GMF, with no significant difference observed between SMF and GMF conditions (Figure 1G). These results underscore the critical role of *fadD* in mediating the inhibitory effects of SMF on bacterial growth. Collectively, our data demonstrate that the inhibitory effect of SMF on growth of *E. coli* colonies is caused by reducing the utilization of LCFA.

### SMF promotes ROS generation in *E. coli* and *in vitro* systems

**Figure 2:**
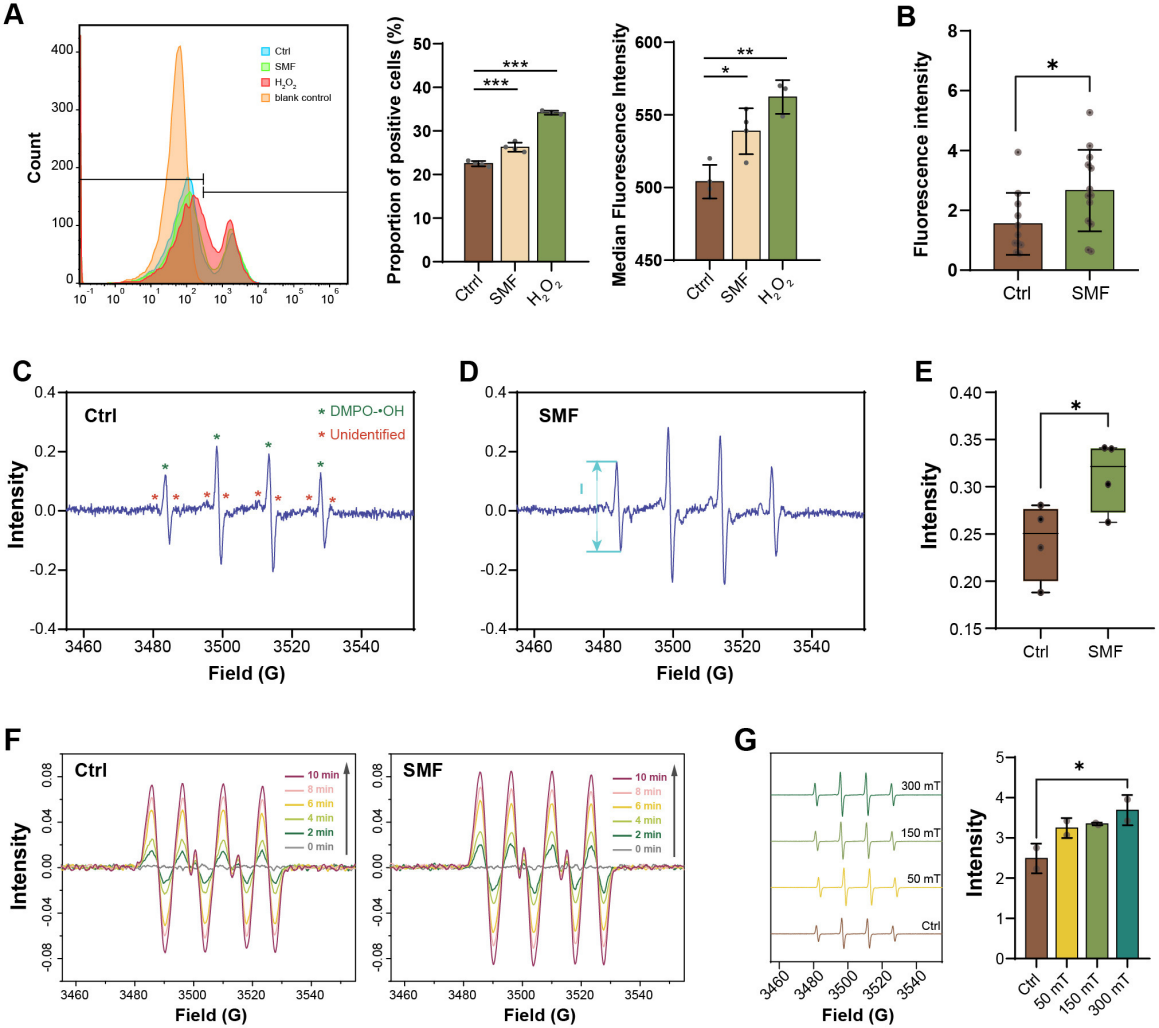
SMF enhances the ROS level in *E. coli* and free radical content *in vitro* reaction systems. **(A)** The proportion of DCFH-DA-staining positive *E. coli* cultured in SMF is higher than that in control group (*n* = 4), Ctrl: GMF control group; SMF: 250 mT SMF-treatment group; H_2_O_2_: 8 μM H_2_O_2_ treatment group; blank control: unstained group. **(B)** Fluorescence intensity of WT/pE-HyPer strain cultured in SMF is higher than that cultured in GMF (*n* > 10). **(C–E)** EPR spectrum of radicals in *E. coli* cultured in SMF (Mag) exhibited higher radical signals (“peak-to-peak”, from maximum to minimum, denoted by I) than those cultured in GMF (Ctrl) (*n* = 4). **(F)** EPR spectrum of radicals of the XO/HX system treated with (Mag) or without (Ctrl) SMF. **(G)** EPR spectrum of radicals of the UV/H_2_O_2_ system treated with varying magnetic flux density. Data shown as mean ± SD. Statistical analysis was performed by student *t*-test, or one-way ANOVA followed by Tukey HSD test. *p < 0.05; **p < 0.01; ***p < 0.001.

ROS are inevitably generated through the electron transport chain (ETC; Agrawal et al., 2017; Jaswal et al., 2020), and have been reported to correlate with magnetobiological effects (Okano, 2008). To investigate whether the inhibition of LCFA degradation is linked to ROS levels under SMF, we employed multiple approaches to quantify ROS in *E. coli*. First, total ROS levels were measured using the fluorescent probe DCFH-DA, which is one of the most widely used probes for ROS detection. Our results showed a significant increase in DCFH-DA-stained cells under SMF compared to the control (Ctrl), indicating elevated ROS production (Figure 2A). Since DCFH-DA is not specific for a particular type of ROS (Zielonka et al., 2012; Murphy et al., 2022), we then utilized the genetically encoded hydrogen peroxide (H_2_O_2_) biosensor HyPer, which incorporates a circularly permuted yellow fluorescent protein (cpYFP) into the H_2_O_2_-sensitive regulatory domain of the bacterial transcription factor OxyR (Belousov et al., 2006). Fluorescence intensity detected by HyPer was significantly higher in SMF-exposed bacteria compared to GMF controls (Figure 2B), confirming increased H_2_O_2_ production under SMF. To further corroborate these findings, we employed electron paramagnetic resonance (EPR) spectroscopy with a radical trapping agent DMPO to quantify the level of radical species. Our results demonstrated that the EPR spectrum of bacterial solution contained a prominent 1:2:2:1 quartet, indicative of the hydroxyl adduct of DMPO-OH, along with several unidentified signals. Notable, the bacteria cultured under SMF exhibited higher radical signals than those cultured in GMF, confirming that SMF treatment enhances cellular free radical levels (Figures 2C–E).

Additionally, we utilized the xanthine oxidase (XO)/hypoxanthine (HX) system to investigate the effects of SMF on free radical reactions. The EPR spectrum obtained from the XO/HX system showed the emergence of DMPO-OOH adduct signals at 2 min, which increased over the next 8 min. In the presence of SMF, these radical signals were further elevated, suggesting that SMF influences free radical reactions (Figure 2F). However, this system could not exclude the possibility that SMF affects radical production via enzymatic activity. To solve this issue, we utilized the protein-free UV/H_2_O_2_ system to evaluate the effect of SMF on radical (hydroxyl radical, •OH) generation. The EPR spectrum recorded under varying SMF intensities (50, 150, and 300 mT) demonstrated a dose-dependent increase in radical signals, with the highest signal observed at 300 mT (Figure 2G), suggesting that SMF promotes ROS production in an enzymatic independent manner. Collectively, these results demonstrate that the rate of ROS generation is accelerated in SMF.

### H_2_O_2_ treatment mimics effects of SMF on *E. coli* growth and gene expression

**Figure 3:**
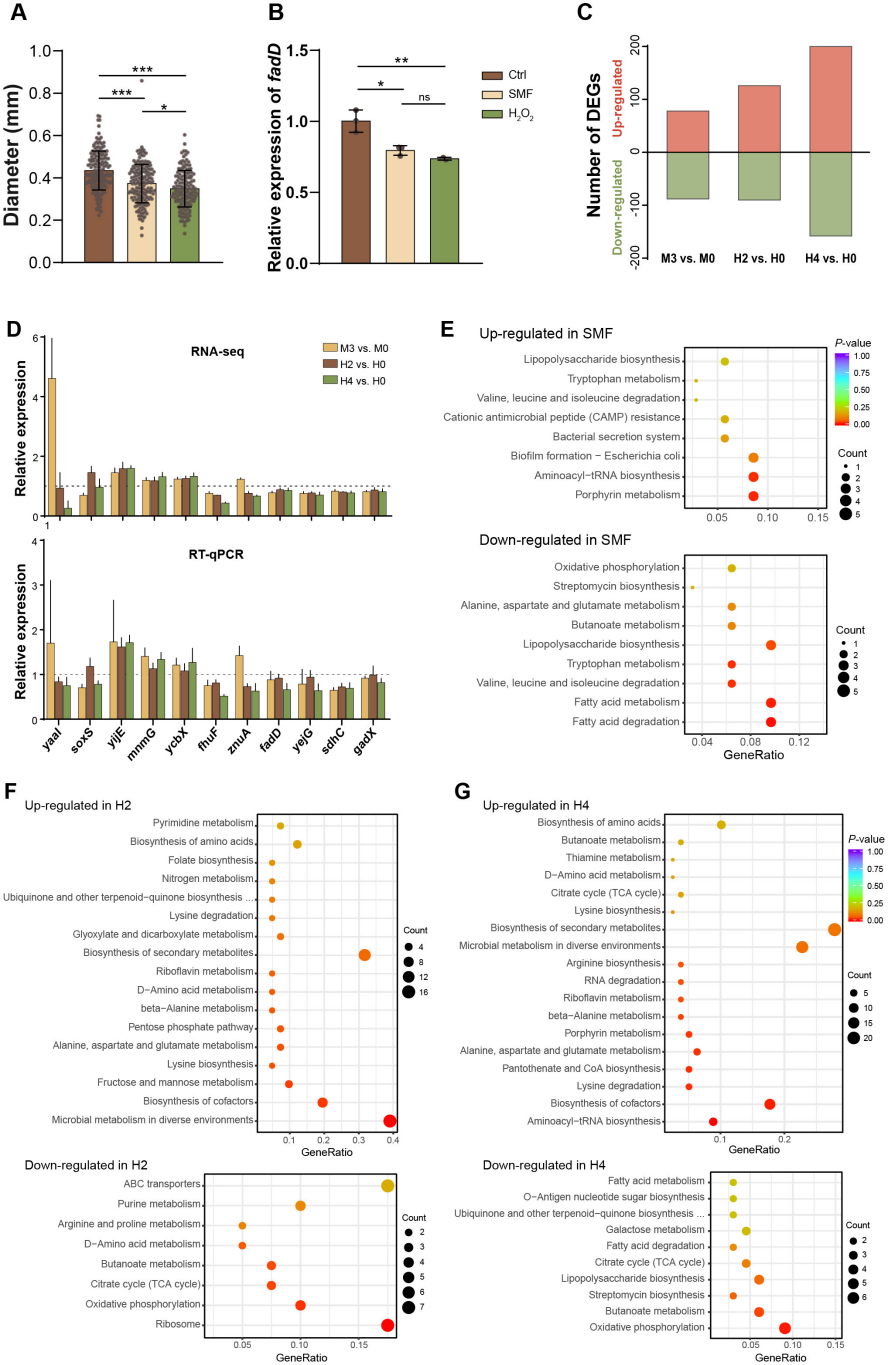
H_2_O_2_ mimics effects of SMF on *E. coli* growth and gene expression. **(A, B)** Represent the CFU diameter and *fadD* expression of bacteria grown under three conditions: Ctrl, SMF and H_2_O_2_, respectively. Data shown as mean ± SD. Statistical analysis was performed by one-way ANOVA followed by Tukey HSD test, n > 50 in **(A)**, n = 3 in **(B)**. Significant differences are indicated by asterisks with ^ns^*p* > 0.05; *p < 0.05; **p < 0.01; ***p < 0.001. **(C)** DEGs of three comparative combinations: M3 vs. M0, H2 vs. H0, and H4 vs. H0. M0 and M3 represent GMF control and 300 mT SMF-treated group respectively, while H0 H2 and H4 represent 0, 2, 4 μM H_2_O_2_-treated group (*n* = 4). **(D)** Validation of RNA-seq data by RT-qPCR. **(E–G)** KEGG analysis of the DEGs induced by SMF-treatment (M3), 2 μM H_2_O_2_ (H2), and 4 μM H_2_O_2_ (H4).

To investigate whether the effect of H_2_O_2_ on bacterial growth is similar to that of SMF, we added H_2_O_2_ (4 μM) to the solid growth media. The results showed that bacterial colonies grown on H_2_O_2_-containing media exhibited a significant reduction compared to those grown on control media, mirroring the growth inhibition observed under SMF (Figure 3A). Real-time quantitative PCR (qPCR) analysis revealed that *fadD* gene expression was downregulated in *E. coli* treated with H_2_O_2_, consistent with the transcriptional changes observed under SMF (Figure 3B). These findings suggests that H_2_O_2_ and SMF induce similar physiological responses in *E. coli*, likely through overlapping mechanisms involving oxidative stress.

To further explore whether the two treatments, SMF and H_2_O_2_, share a similar influence on the global gene expression, we performed RNA-sequencing (RNA-seq) on samples treated with GMF (M0), 300 mT SMF (M3), 0 μM H_2_O_2_ (H0), 2 μM H_2_O_2_ (H2), and 4 μM H_2_O_2_ (H4), with four independent biological replicates. A total of 4,270 expressed genes were identified from all samples (Supplementary Table S2). There were 166, 216, and 358 differentially expressed genes (DEGs) identified from three comparative combinations: M3 vs. M0, H2 vs. H0, and H4 vs. H0, respectively, with a significance threshold of *p* < 0.05 (Figure 3C, Supplementary Table S5). These results indicate that both SMF and H_2_O_2_ treatments in this study are mild environmental stresses that did not widely modulate bacterial gene expression. To verify the transcriptomic data, we employed qPCR to assess the mRNA changes of 11 selected genes. Overall, our results were in agreement with the transcriptomic data, reinforcing the findings of our RNA-seq analysis (Figure 3D).

To elucidate whether SMF and H_2_O_2_ share similar mechanisms in inhibiting bacterial growth, we first independently analyzed biological effects of these treatments using the identified DEGs. Among the 166 DEGs regulated by SMF, 88 were downregulated while 78 were upregulated (Supplementary Table S3). Functional enrichment analysis using the KEGG revealed that the downregulated DEGs were primarily enriched in pathways related to fatty acid metabolism, lipopolysaccharide biosynthesis and oxidative phosphorylation (Figure 3E). For example, key genes involved in fatty acid degradation, such as *fadB, fadD, fadI*, and *fadH*, were downregulated under SMF. Additionally, genes encoding components of the electron transport chain (*sdhC, cyoA*), iron-sulfur (Fe-S) clusters assembly machinery (*iscR, iscS, iscU, iscA*), and the FtsZ ring stabilization factor essential for bacterial division (*zapC*) were also repressed. Notably, the *SoxS* gene, which encodes a transcriptional activator of the superoxide response, were downregulated in the M3 samples, suggesting a suppression of oxidative stress defense mechanisms under SMF. Conversely, the upregulated DEGs were enriched in pathways related to porphyrin metabolism, aminoacyl-tRNA biosynthesis, and biofilm formation (Figure 3E). Among these, *fliN* (a flagellar motor switch protein) and genes involved in cysteine metabolism (*cyuA, yijE*) were significantly upregulated. These findings collectively indicate that SMF triggers oxidative stress, leading to the downregulation of metabolic pathways essential for growth and the upregulation of stress-response mechanisms.

In the H_2_O_2_-treated groups, the transcriptional responses were concentration-dependent. In the H2 group (2 μM H_2_O_2_), 90 DEGs were downregulated and 126 were upregulated. The downregulated DEGs were primarily enriched in ribosome, oxidative phosphorylation, and the citrate cycle pathways, while the upregulated DEGs were enriched in microbial metabolism in diverse environments, amino acid metabolism, biosynthesis of cofactors, fructose and mannose metabolism, and amino acid metabolism (Figure 3F). At a higher H_2_O_2_ concentration (4 μM, H4 group), the number of DEGs increased to 358, with 158 downregulated and 200 upregulated. The downregulated DEGs were enriched in oxidative phosphorylation, butanoate metabolism, and lipopolysaccharide biosynthesis pathways, whereas the upregulated DEGs were mainly involved in aminoacyl-tRNA biosynthesis, biosynthesis of cofactors, amino acid metabolism, pantothenate and CoA biosynthesis, and microbial metabolism in diverse environments (Figure 3G). Thus, these results indicate that bacteria adapt to low concentrations of H_2_O_2_ through downregulation of central metabolism and growth-related pathways and upregulation of stress-response pathways, but to a higher concentration of H_2_O_2_ via prevention of further oxidative damage. Further analysis showed that 86 DEGs were shared in both comparative combinations of H2 vs. H0 and H4 vs. H0. Among these DEGs, many of them are responsible for ROS scavenging and regulation of intracellular redox levels, as well as genes related to energy metabolism. For instance, three genes (*katG, trxC*, and *sufB*) involved in hydrogen peroxide detoxification were upregulated (Supplementary Table S5), and 12 genes involved in cellular respiration under oxidative stress conditions were downregulated (Supplementary Table S4). Notably, *katG*, encoding catalase-peroxidase that rapidly degrade extracellular H_2_O_2_ and promote bacterial survival, was upregulated in both H2 and H4 groups, highlighting its critical role in H_2_O_2_ detoxification. Additionally, a large number of upregulated genes are related to amino acid metabolism (*dapF, lysA, gabD, gabT, glnA, argG*) and biosynthesis of cofactors, suggesting a robust stress response to oxidative damage. Several genes involved in aminoacyl-tRNA biosynthesis (*serS, gltX, glnS, lysS, argS, cysS*, and *metG*) were only upregulated by 4 μM H_2_O_2_ (H4), suggesting that the translation process of mRNA is promoted, as the drastic changes in cell metabolism require more proteins or enzymes. Collectively, these results indicate that bacteria adapt to H_2_O_2_-induced oxidative stress by downregulating central metabolic pathways and upregulating stress-response and detoxification mechanisms.

**Figure 4:**
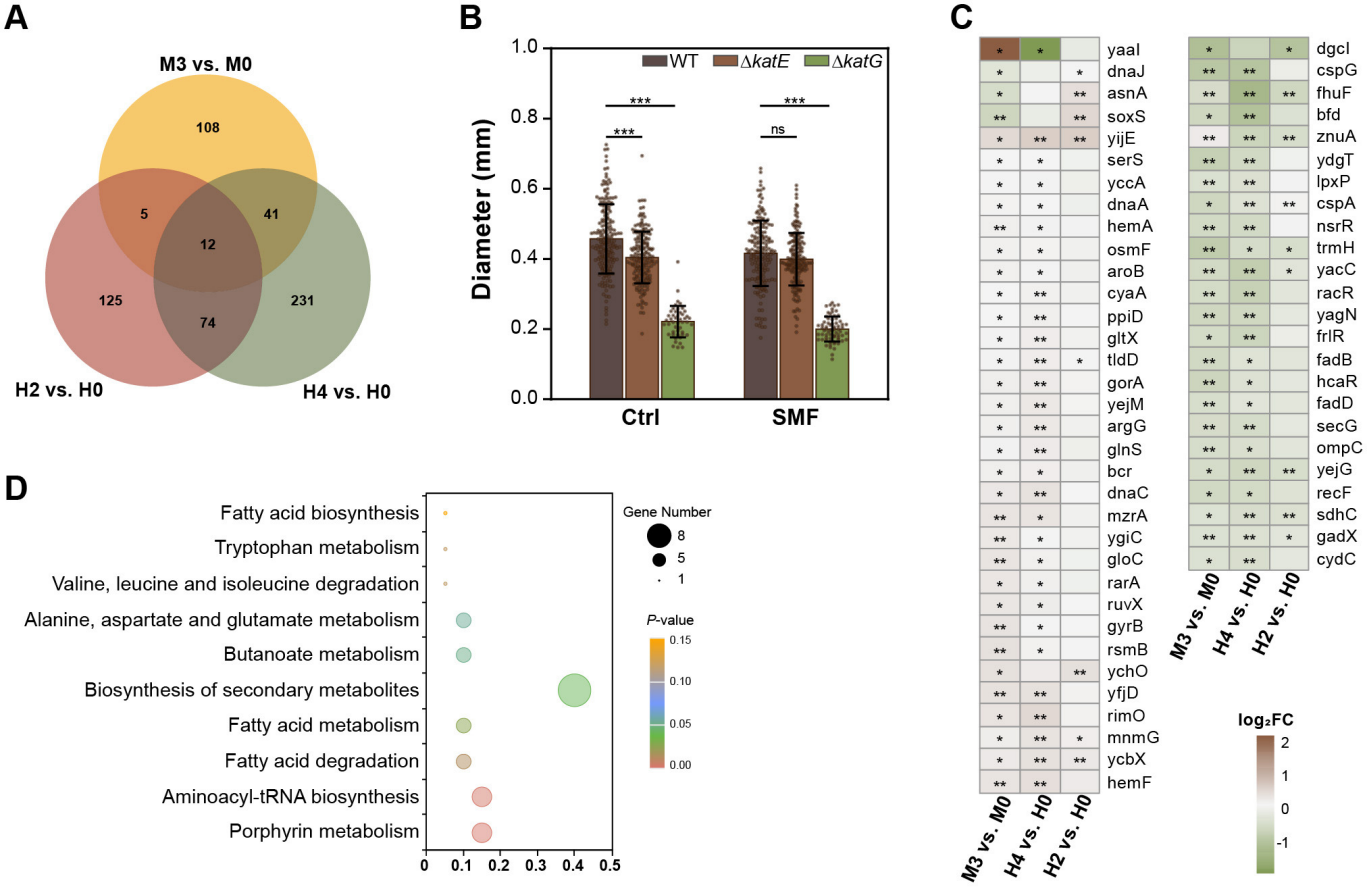
Comparative analysis of transcriptomic profiles of SMF-, 2 μM and 4 μM H_2_O_2_-treated bacteria. **(A)** The Venn diagram DEGs of three comparative combinations: M3 vs. M0, H2 vs. H0, H4 vs. H0. **(B)** Effect of *katE* or *katG* mutations on bacterial growth. **(C)** 58 DEGs are shared by M3 vs. M0 and H2 vs. H0 or H4 vs. H0. **(D)** KEGG analysis of the shared DEGs of three comparative combinations. Data are shown as mean ± SD. Statistical analysis was performed by one-way ANOVA followed by Tukey HSD test (n > 50). ^ns^*p* > 0.05; *p < 0.05; **p < 0.01; ***p < 0.001.

To identify common mechanisms underlying the effects of SMF and H_2_O_2_, we analyzed transcriptomic profiles from comparative combinations of M3 vs. M0, H2 vs. H0, and H4 vs. H0. A total of 12 DEGs were shared across all three treatments, including 4 upregulated and 6 downregulated genes, as well as 2 genes with inconsistent changes (Supplementary Table S3). In addition, 10.24 and 31.93% of SMF-regulated DEGs were overlapped with those H2- and H4-treated groups, respectively, suggesting that the transcriptomic profile of SMF is more similar to that of H4, compared to that of H2 (Figures 4A, C). KEGG enrichment analysis revealed that these overlapping DEGs were mainly enriched in pathways related to porphyrin metabolism, aminoacyl-tRNA biosynthesis, fatty acid metabolism, and the biosynthesis of secondary metabolites (Figure 4D). Interestingly, we found four commonly down-regulated genes encoding transcriptional regulators, namely NsrR that responds to nitric oxide levels to regulate genes involved in oxidative stress responses, RacR that regulates expression of genes associated with stress responses or metabolism, FrlR that is responsible for the regulation of fructosyltransferase activity (carbohydrate metabolism), and HcaR that regulates genes involved in the metabolism of hydroxycinnamic acid. These results suggest that the transcriptional factors play an important role in SM- and H_2_O_2_-inhibited bacterial growth. Furthermore, key genes in fatty acid metabolism (*fadD, fadB*), the tricarboxylic acid (TCA) cycle (*sdhC*), and aerobic respiration (*cydC*) were significantly downregulated, further supporting the notion that SMF and H_2_O_2_ inhibit bacterial growth by disrupting central metabolic pathways. Additionally, we found that several tRNA-related transcripts were upregulated, suggesting alterations in translational efficiency under oxidative stress. The upregulation of *yijE*, a cystine efflux transporter, further indicates an increased oxidative status in SMF- and H_2_O_2_-treated bacteria (Yamamoto et al., 2015).

To validate the role of oxidative stress in SMF-induced growth inhibition, we constructed knockout mutants of *katE* and *katG*, which encodes catalase-peroxidase enzymes. Our data indicated that two knockout strains, Δ*katE* and Δ*katG*, both exhibited reduced growth under GMF conditions (Figure 4B), indicating that increased H_2_O_2_ concentration inhibits bacterial growth. Interestingly, the growth of Δ*katG* was heavily inhibited, suggesting that *katG* plays a major role in scavenging ROS in bacteria. Under SMF, Δ*katE* strain had the same diameter of colonies as WT, indicating that *katE* might mediate the inhibitory effect of magnetic fields on the growth of *E. coli*. Taken together, our results suggest that elevated intracellular H_2_O_2_ levels contribute to the inhibitory effects observed in *E. coli* cultured in SMF.

### Superoxide mediates SMF-induced growth inhibition

**Figure 5:**
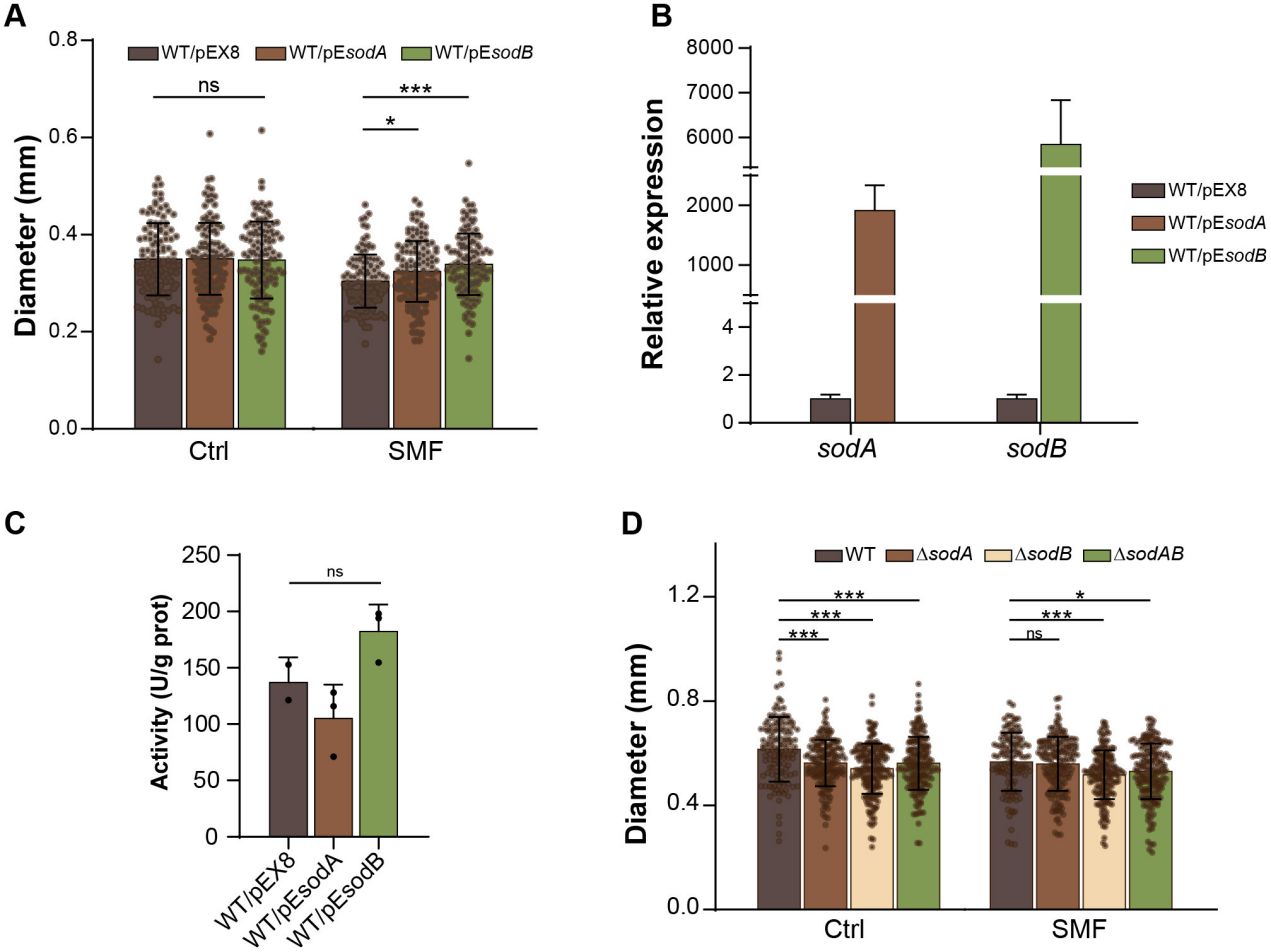
Superoxide anion mediates SMF-induced growth inhibition. **(A)** Overexpression of *sodA* or *sodB* suppresses SMF-inhibited growth of *E. coli*. **(B)** Relative expression levels of *sodA* and *sodB* in transgenic strains under GMF control. **(C)** The activities of SOD in *sodA* and *sodB* overexpressed strains. **(D)** Effect of *sod* mutations on bacterial growth. Data are shown as mean ± SD. Statistical analysis was performed by one-way or two-way ANOVA followed by Tukey HSD test. ^ns^*p* > 0.05; *p < 0.05; ***p < 0.001.

To confirm the role of superoxide anion in SMF-induced growth inhibition, we overexpressed *sodA* and *sodB* genes, encoding Mn- and Fe-containing SOD, respectively, which have been demonstrated to play an important role in conversion of superoxide anion radicals into hydrogen peroxide in *E. coli* (Tsuji et al., 2003; Baez and Shiloach, 2017). Our findings revealed that overexpression of both *sodA* (WT/pE*sodA*) and *sodB* (WT/pE*sodB*) effectively rescued the slow-growth phenotype of bacteria under SMF, and *sodB* overexpression conferred a more pronounced restorative effect than *sodA* (Figure 5A). qPCR confirmed substantial upregulation of *sodB* and *sodA* in the respective strains (Figure 5B). Enzymatic analysis showed that the activity of SOD in the WT/pE*sodB* strain was also increased, compared to WT (Figure 5C). However, the enzymatic activity of the WT/pE*sodA* strain was decreased. This disparity likely arises from intrinsic differences in catalytic efficiency between Mn-SOD (SodA) and Fe-SOD (SodB) isoforms, combined with potential disruption of endogenous redox regulatory networks (e.g., *soxRS* system) upon plasmid-mediated overexpression. These results suggest that scavenging superoxide anion radicals can alleviate the inhibition of bacterial growth in SMF. Furthermore, single and double mutants of *sodA* and *sodB* exhibited reduced growth compared to WT under GMF conditions (Figure 5D), indicating that superoxide anion accumulation inhibits bacterial growth. Under SMF, the Δ*sodB* single and Δ*sodAB* double mutants grew more slowly than WT, whereas Δ*sodA* strain had the same diameter of colonies as WT, suggesting that *sodB* plays a major role in scavenging ROS in bacteria. Collectively, our results demonstrate that superoxide anion mediates the inhibitory effects of SMF on bacterial growth.

## Discussion

In this study, we demonstrate that exposure to SMF elevates the ROS level in *E. coli*, inducing oxidative stress and significantly inhibiting bacterial growth. Physiological and genetic analyses suggest that the observed growth inhibition in SMF-treated *E. coli* is primarily due to impaired LCFA degradation. Transcriptomic profiling further revealed that genes involved in fatty acid metabolism, the TCA cycle and ETC are consistently downregulated under both SMF and H_2_O_2_ treatments. Moreover, genetic manipulations aimed at mitigating oxidative stress, such as overexpression of superoxide dismutase (*sodB*), significantly alleviated SMF-induced growth inhibition. These findings highlight a critical mechanism by which oxygen acts as a magnetic target, initiating oxidative signaling and modulating metabolic pathways to enable bacterial adaptation to SMF exposure.

SMF have been shown to exert diverse effects on microbial physiology, such as microbial central metabolism and stress responses. For instance, SMF can enhance microbial fermentation processes by increasing biomass and product yields, as observed in glutathione production by yeast (Santos et al., 2010; Akarca and Denizkara, 2024). This improvement is likely due to the modulation of microbial metabolic pathways, favoring the synthesis of desired products while minimizing unwanted by-products. SMF can also enhance microbial growth by increasing cell division rates and biomass accumulation, leading to accelerated growth and higher population densities (Letuta et al., 2017; Masood, 2017). However, under specific conditions, SMF can inhibit microbial growth. For instance, short-term exposure to SMF (30 min at intensities ranging from 45 mT to 3500 mT) can significantly reduce *E. coli* viability due to cell surface damage (Ji et al., 2009), while prolonged exposure (4-h at 100 mT) can inhibit bacterial adhesion and colony formation by disrupting cell membranes (Bajpai et al., 2012). Furthermore, SMF has been shown to enhance microbial stress resistance and improve survival under adverse conditions by activating stress response pathways, including heat shock and antioxidant defenses, which help to maintain cellular homeostasis (Tan et al., 2020; Wang et al., 2020; Cardoso et al., 2024).

The regulation of ROS production by SMF is well recognized, although its effects can be either positive or negative, depending on the intracellular and extracellular stimuli. For example, Vergallo *et al*. reported that 24-h exposure to SMF enhances ROS production, protecting neuroblastoma cells against cisplatin (Cis-DichloroDiammine Platinum II, *cis*Pt)-induced apoptosis (Vergallo et al., 2014). Similarly, Song et al. demonstrated that moderate SMF suppresses ovarian cancer metastasis by increasing ROS-mediated oxidative stress (Song et al., 2021). In contrast, SMF has also been shown to reduce oxidative stress, improving wound healing and alleviating diabetic complications (Feng et al., 2022), as well as delaying the senescence of strawberries by mitigating ROS accumulation (Liu et al., 2023). In our study, we employed multiple approaches, including chemical fluorescent dye (DCFH-DA), genetically encoded fluorescent sensor (HyPer), and EPR spectroscopy to quantify ROS levels, all of which consistently demonstrated that SMF elevates ROS levels in *E. coli* (Figure 2). In addition, EPR analysis also revealed several unidentified signals that increased in SMF-treated bacteria, suggesting a pervasive effect of SMF on cellular radical dynamics. The radical pair recombination theory provides a plausible explanation for the effects of SMF on ROS generation (Montoya, 2016; Usselman et al., 2016; Doktorov and Lukzen, 2023). Molecular oxygen (O_2_) has two unpaired electrons with parallel spins occupying separate molecular orbitals, called a triplet ground state (^3^O_2_) under normal conditions. This spin restriction makes oxygen relatively unreactive. However, when excited by energy from redox-active species or light irradiation, O_2_ can transition to a singlet state (^1^*O*_2_), which has unpaired electrons with opposite spins. The excited ^1^O_2_ is highly reactive and can readily react with organic molecules, such as lipids, proteins, and DNA, contributing to oxidative stress. External magnetic field influence the spin states of oxygen by modulating the magnetic interactions between electronic spins and nuclear magnetic moments, thereby affecting the frequency of singlet-triplet transitions and radical recombination rates (Doktorov and Lukzen, 2023). Our *in vitro* experiments using systems such as XO/HX and UV/H_2_O_2_ demonstrated enhanced radical signals in the presence of external SMF, consistent with the free radical pair recombination theory. These findings suggest that SMF promotes the formation of radical pairs, leading to increased ROS production, including superoxide anions (${\text{O}}_{2}^{•-}$) and hydroxyl radicals (•OH) in an enzymatic independent manner. Recently, Yang *et al*. reported an unprecedented MF dependence of the reactivity of singlet oxygen (^1^O_2_) toward electron-rich substrates (S), where the formed [S^•+^ ${\text{O}}_{2}^{•-}$] radical pairs act as a basis for MF to affect molecular redox events in biological systems (Yang et al., 2024). Thus, SMF-induced ROS production can be a universal mechanism to help us understand bioeffects of SMF in cells.

In the cellular context, the effects of SMF on radicals are more intricate due to the complex and diverse free radical reactions and the regulatory mechanisms that maintain intracellular homeostasis. To explore the effects of SMF on cellular free radical reactions, we conducted RNA-seq analyses to compare the transcriptional responses of *E. coli* to SMF and H_2_O_2_ treatments. The results revealed significant overlap in differentially expressed genes (DEGs) between the two treatments, suggesting a potential shared regulatory mechanism. In fact, H_2_O_2_ is known to function as a signal to initiate signaling pathways by direct interactions with receptors or by oxidation of various biomolecules including signal transduction proteins and transcription factors (Burdon, 1995; Okano, 2008). Furthermore, different concentrations of H_2_O_2_ induce distinct transcriptional responses, with higher concentrations eliciting more pronounced changes in gene expression. Analysis of overlapping DEGs indicated that, in addition to the upregulation of genes involved in H_2_O_2_ detoxification, modulation of genes associated with central metabolism is also conserved response to oxidative stress in *E. coli*. Interestingly, several DEGs are annotated as being regulated by the ArcAB two-component system, which senses oxygen consumption and regulates central metabolic pathways. These genes including *sdhC* (responsible for succinate dehydrogenase), and *fadD*/*B* (for fatty acid degradation) were down-regulated, suggesting that cellular metabolism related to fatty acid and the TCA cycle were inhibited under SMF. Given the close linkage between the cellular redox state and metabolism, the involvement of ArcAB in maintaining redox homeostasis under stress conditions is anticipated (Brown et al., 2022). These findings suggest that the bioeffects of SMF are closely associated with metabolic modulation in ROS resistance-related pathways. Additionally, the overlapping DEGs include genes involved in non-coding RNA (ncRNA) metabolic processes. For instance, genes such as *glnS* (glutamine–tRNA ligase), *gltX* (glutamate–tRNA ligase), *ruvX* (putative pre-16S rRNA nuclease), and *serS* (serine–tRNA ligase) were up-regulated in both SMF and 4 μM H_2_O_2_ treatment groups, suggesting that *E. coli* may manipulate gene expression and protein translation to cope with stress induced by SMF and H_2_O_2_. In summary, the inhibitory effects of SMF on *E. coli* growth and metabolism are partially mediated through the oxidative stress pathway. However, the presence of numerous specific DEGs in the SMF-treated group suggests additional mechanisms, independent of H_2_O_2_, contribute to SMF-induced growth inhibition. Beyond the overlapping DEGs shared between SMF and H_2_O_2_ treatments, more DEGs are specifically induced by SMF treatment. One notable example is the downregulation of the *isc* operon, which is responsible for the assembly of Fe-S clusters. Within this operon, *iscA*, also known as *MagR* that plays a key role in animal magnetoreception, is involved in the filamentous shape change in response to iron accumulation in *E. coli* (Lu et al., 2008; Wei et al., 2024). Additionally, the expression of the *isc* operon is also regulated by the intracellular redox state. These results suggest that a multi-faceted process involving the redox state, carbon metabolism, iron-cluster assembly is tightly associated with the bioeffects of SMF on *E. coli*.

While H_2_O_2_ was identified as a significant ROS involved in SMF-induced growth inhibition, other members such as superoxide anions, also contribute to the inhibitory effects. Our genetic studies on superoxide dismutase genes (*sodA* and *sodB*) revealed that overexpression of *sodA* or *sodB* alleviated growth inhibition under SMF, while knockout mutants (Δ*sodB*) exhibited heavier inhibition of bacterial growth under both GMF and SMF conditions. However, knockout of *sodA* had no significant impact on SMF-induced growth inhibition, suggesting that *sodA* plays a relatively minor role in the bacterial response to SMF, compared to *sodB*, at least under the experimental conditions tested. Also, the total SOD activities of the overexpression strain confirmed the higher activity of SODB. These findings collectively indicate that *sodB* is the primary superoxide dismutase responsible for detoxifying superoxide anions and alleviating oxidative stress in *E. coli* under SMF exposure. This *sodB*-centric mechanism extends beyond *E. coli*. In *Pseudomonas aeruginosa, sodB*-deficient mutants exhibited more sensitivity to 200 mT SMF, with significantly reduced survival rates and antioxidant enzyme activities (SOD, catalase) compared to wild-type strains. Furthermore, double mutants lacking both *sodB* and *sodM* (Mn-SOD) showed the most severe lipid peroxidation and growth defects under SMF, underscoring the indispensable role of *sodB* in bacterial magnetic stress adaptation (Hanini et al., 2017). The critical role of superoxide detoxification in magnetic field responses was also highlighted by mammalian studies, where scavenging mitochondrial ${\text{O}}_{2}^{•-}$ via SOD2 overexpression abolished magnetic field-induced metabolic improvements (Carter et al., 2020). This evolutionary parallel suggests that superoxide-mediated magnetic sensing may represent a conserved mechanism across biological systems, though implemented through distinct SOD isoforms. The differential roles of *sodA* and *sodB* highlight the specificity of enzymatic responses to oxidative stress and provide insights into the molecular mechanisms by which bacteria adapt to SMF-induced perturbations in redox balance.

LCFA are a tremendous source of metabolic energy, as their degradation generates a large number of reduced cofactors that drive ATP synthesis via ETC. Under normal conditions, electrons in the ETC are ultimately transferred to molecular oxygen (O_2_) to form H_2_O; however, possible electron leakage during oxidation-reduction cycles can lead to the adventitious collision of free electrons with O_2_, causing ROS production (Jaswal et al., 2021). It has been reported that the utilization of LCFA generates higher ROS levels than other carbon sources, like glucose, acetate, or succinate (Agrawal et al., 2017). To balance the benefits of LCFA degradation with the cost of redox stress, cells employ feedback mechanisms to regulate ROS production (Olsen et al., 2013; Nakamura et al., 2014; He et al., 2023). For example, reducing intracellular H_2_O_2_ levels has been shown to improve L-lysine fermentation that uses fatty acid as raw materials in *E. coli* (Doi et al., 2014), while increased hydroxyl radicals under stress conditions upregulate the expression of genes involved in fatty acid and glycerolipid biosynthesis, promoting lipid accumulation in *Chlorella pyrenoidosa* (Zhang et al., 2019). Consistently, our findings indicate that SMF exposure leads to increased ROS levels and inhibition of LCFA degradation, as evidenced by the downregulated expression of *fad* genes, which encode the enzymes required for β-oxidation. Experimental results with supplementation of fatty acids of different chain lengths also suggest that the *fadD* gene plays a crucial role in SMF-induced growth inhibition of *E. coli*. Additionally, the role of *fadD* was further validated by constructing overexpression and knockout strains. Overexpression of *fadD* restored the growth phenotype under SMF conditions, while the knockout mutant Δ*fadD* exhibited diminished growth but was insensitive to SMF, indicating that *fadD* downregulation is a critical factor in SMF-induced growth inhibition. This suggests that enhancing LCFA degradation capacity can mitigate the inhibitory effects of SMF, highlighting *fadD* as a potential target for modulating bacterial responses to SMF.

**Figure 6:**
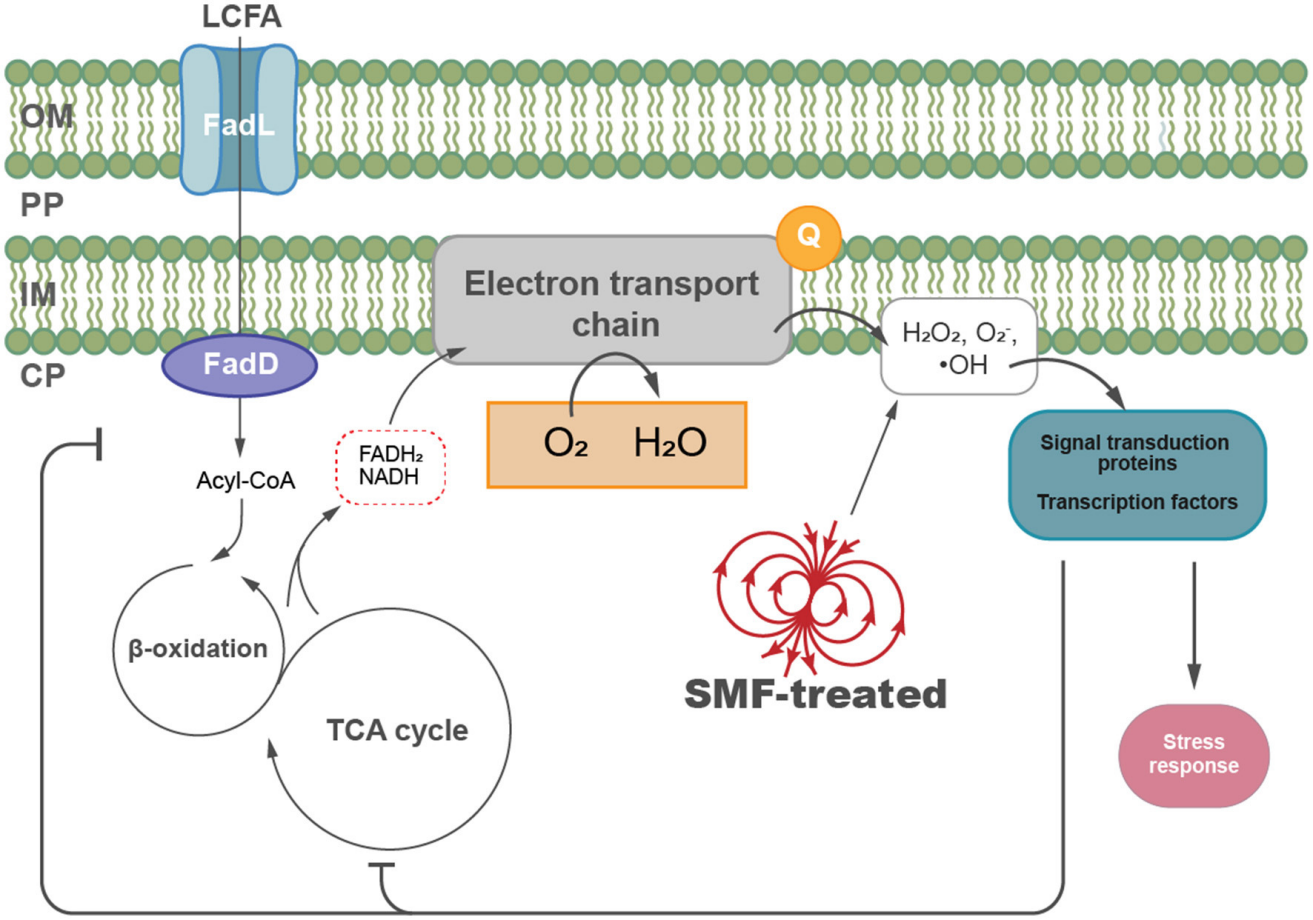
A proposed model illustrating the biological effects of SMF on *E. coli* growth. SMF exposure increases intracellular ROS levels, such as hydrogen peroxide and superoxide anions, inducing oxidative stress and modulation of key metabolic pathways, including fatty acid metabolism and central carbon metabolism in *E. coli*. Key components include long-chain fatty acid (LCFA), the outer membrane (OM), periplasm (PP), inner membrane (IM), and cytoplasm (CP).

Based on our findings, we propose a model to summarize the effects of SMF on *E. coli* (Figure 6). SMF exposure increases intracellular ROS levels, such as hydrogen peroxide and superoxide anions, inducing oxidative stress and disrupting cellular homeostasis. To adapt to this stress, *E. coli* modulates key metabolic pathways, including fatty acid metabolism and central carbon metabolism, to restore redox balance and maintain energy production. These metabolic adjustments are critical for mitigating oxidative damage and sustaining cellular viability under SMF conditions. This model highlights the complex interplay between oxidative stress and metabolic regulation, demonstrating the ability of *E. coli* to dynamically adjust its internal processes in response to environmental changes induced by SMF. Understanding these mechanisms provides valuable insights into potential strategies for managing bacterial growth in magnetic field environments.

## Data Availability

The transcriptomic data presented in the study are deposited in the SRA repository, accession number PRJNA1276862.
